# Information to Improve Public Perceptions of the Food and Drug Administration (FDA’s) Tobacco Regulatory Role

**DOI:** 10.3390/ijerph15040753

**Published:** 2018-04-14

**Authors:** Amira Osman, Sarah D. Kowitt, Paschal Sheeran, Kristen L. Jarman, Leah M. Ranney, Adam O. Goldstein

**Affiliations:** 1 Lineberger Comprehensive Cancer Center, University of North Carolina, Chapel Hill, NC 27599, USA; jkristen@email.unc.edu (K.L.J.); adam_goldstein@med.unc.edu (A.O.G.); 2 Department of Health Behavior, Gillings School of Global Public Health, University of North Carolina, Chapel Hill, NC 27599, USA; kowitt@email.unc.edu; 3 Department of Psychology and Neuroscience, University of North Carolina, Chapel Hill, NC 27599, USA; psheeran@email.unc.edu; 4 Department of Family Medicine, University of North Carolina, Chapel Hill, NC 27599, USA; Leah_Ranney@unc.edu

**Keywords:** FDA, tobacco regulations, source credibility, perceptions

## Abstract

While the Food and Drug Administration (FDA) has had regulatory authority over tobacco products since 2009, public awareness of this authority remains limited. This research examines several broad types of information about FDA tobacco regulatory mission that may improve the perceptions of FDA as a tobacco regulator. Using Amazon Mechanical Turk, 1766 adults, smokers and non-smokers, were randomly assigned to view a statement about FDA regulatory authority that varied three information types in a 2 × 2 × 2 between subjects experimental design: (1) FDA’s roles in regulating tobacco (yes/no); (2) The scientific basis of regulations (yes/no); and (3) A potential protective function of regulations (yes/no). Using factorial ANOVA, we estimated the main and interactive effects of all three types of information and of smoking status on the perceptions of FDA. Participants that were exposed to information on FDA roles reported higher FDA credibility and a greater perceived knowledge of FDA than those who did not. Exposure to information about the scientific basis of regulations led to more negative views of the tobacco industry. Participants who learned of the FDA’s commitment to protecting the public reported higher FDA credibility and more positive attitudes toward regulations than those who did not learn of this commitment. We observed no significant interaction effects. The findings suggest that providing information about the regulatory roles and protective characterization of the FDA’s tobacco regulatory mission positively influence public perceptions of FDA and tobacco regulations.

## 1. Introduction 

The Tobacco Control Act and the deeming rule give the Food and Drug Administration (FDA) a broad authority to regulate all tobacco products, including their manufacture, marketing, and distribution [1,2]. Under this authority, the FDA’s Center for Tobacco Products (CTP) examines and makes determinations on new tobacco products that seek market authorization, conducts rigorous compliance and enforcement programs to ensure that tobacco industry and others (e.g., distributors, retailers) follow the law, educates the public about the dangers of tobacco products, and funds and conducts research to support and establish regulations concerning tobacco products that are appropriate for the protection of the public health [1].

To achieve its mission, the FDA’s CTP is responsible for informing the public and various stakeholders (e.g., tobacco companies, manufacturers, distributers, retailers, and advocacy groups) about the FDA’s authority over tobacco products. Public perceptions of FDA are likely to influence the public’s and stakeholder’s attitudes towards the agency and its tobacco regulations [3]. Perceptions of FDA’s credibility as a tobacco regulator (i.e., its expertise, trustworthiness, and accountability in this role) may also determine receptivity to, and persuasion by, FDA communications about the harms of tobacco products [4]. Research in the United States (US), however, shows moderate levels of trust in government agencies, including those that were tasked to implement tobacco control measures [5,6]. One study found that federal agencies, such as FDA, the Centers for Disease Control (CDC), and the National Institutes of Health (NIH) emerged third (42%) in ratings of trust in online health information resources after personal doctor (59%), and medical universities (48%) [6]. In a 2015 Pew Research Center Poll, only 51% and 71% of the US population reported that they view the FDA and CDC favorably [7]. A more recent study by Kowitt et al., (2017) found that only 43% of US adults reported trust in the federal government and that trust in FDA was moderate (62%) but lower than trust in the CDC (65%) [5]. Research also shows low public awareness of FDA’s tobacco regulatory and communication roles [8,9,10,11]. 

Historically, public opinion about tobacco use has been shaped by promotional and marketing activities of the tobacco industry, which included decades of obscuring the truth about the addictiveness of nicotine and the negative health consequences of tobacco use [12]. Relatedly, tobacco control programs to reduce tobacco consumption have frequently been countered by the tobacco industry in ways designed to maintain its customer base [13,14,15,16]. Many rigorous and effective efforts have been undertaken to counter tobacco industry activities including exposing industry documents, the MSA settlement [17], advertising restrictions, major legislative action (The Family Smoking Prevention and Tobacco Control Act, 2009, The deeming rule) [1,2], and major tobacco prevention public education campaigns (e.g., Truth campaign, Tips from former smokers campaign, Real Cost campaign) that directly support FDA’s mission by educating the public about the dangers of regulated tobacco products [18,19,20]. Despite great accomplishments for tobacco control in the US, industry activities continue to encourage the consumption of harmful, traditional, and new and emerging, tobacco products [12]. In this context, it is important to establish and maintain a public and stakeholder opinion that understands and supports tobacco control measures to curb the tobacco epidemic. 

What types of information would serve to increase awareness about, improve the credibility of, and enhance public attitudes towards tobacco control communications and regulations, such as those that were promoted by the FDA? The present study draws upon research on brand identification [21] in order to test the role of three types of information in improving public perceptions of FDA. The first is information about the agency’s regulatory role: what does the FDA do, and the rationale for its role to safeguard the public’s health. The second is information about the scientific basis of FDA regulations: regulations are derived from scientific evidence. The third is information about the regulation’s protective function. In this study, we examine the effect of one possible example of a protective function statement that describes the FDA’s commitment to protecting Americans by regulations from unlawful tobacco industry activities. 

## 2. Methods 

### 2.1. Participants and Procedures 

Data were collected via Amazon Mechanical Turk (MTurk) from 1766 adults (N = 863 smokers, N = 903 non-smokers), aged 18 to 75 years. Current smokers were defined as those who have smoked at least 100 cigarettes in their lifetime and have smoked every day or some days in the past 30 days. Participants were asked to read a consent form prior to taking the survey and were compensated $1 for completing a 10-min. survey. 

#### Human Subjects Approval Statement 

The study protocol was approved by the Ethics Review Board at the University of North Carolina at Chapel Hill (Study # 17-0138).

### 2.2. Experimental Manipulations 

The survey was programmed and administered using Qualtrics, which is an online survey platform (Qualtrics, Provo, UT; https://www.qualtrics.com) [22]. In a 2 × 2 × 2 between subjects experimental design, participants were randomly assigned to view a statement that varied three types of public information: (1) Information on FDA’s role—The FDA regulates the manufacture, marketing, and distribution of tobacco to safeguard people’s health; (2) Information on the scientific basis of FDA regulations—FDA regulations are based on scientific evidence; and, (3) Information on a potential protective function of FDA regulations—FDA regulations are designed to protect Americans from the activities of the tobacco industry. This resulted in eight experimental conditions, one of which provided no information about the FDA (see Table 1). We used the randomizer tool in Qualtrics, which uses a random number generator, to assign participants to conditions as they enter the study. The randomizer also allowed for an even allocation of participants across conditions [23]. Participants were asked to read the statement and then answer questions.

### 2.3. Questionnaire

#### 2.3.1. Pre-Test Questions

Prior to viewing the experimental statement, participants indicated their overall trust in the federal government: “How much trust do you have in the Federal government? (4-point scale; 0 = none at all, 4 = a great deal) and their knowledge of FDA’s role in regulating tobacco via four questions: “Do you think the FDA regulates how cigarettes and other tobacco products are (1) made (2) advertised (3) and sold in stores?” and, (4) “Do you think the FDA communicates the risks of cigarettes and other tobacco products to the public?”. Reponses were 1 = Yes, 0 = No. 

#### 2.3.2. Post-Test Questions 

After exposure to the experimental statement, we assessed four constructs.

(1) Perceived knowledge of FDA’s tobacco regulatory roles was assessed by three questions adapted from previous studies [24,25], (1) “How much information do you feel you have about the FDA’s role in regulating tobacco?”(5-point scale; 0 = not at all to 4 = a great deal) [25]; (2) “How knowledgeable do you feel you are about the FDA’s role in regulating tobacco?” (5-point scale; 0 = not at all knowledgeable to 4 = extremely knowledgeable) [25]; and, (3) “To what extent do you agree or disagree with the following statement: ‘When it comes to how FDA regulates tobacco, I really don’t know a lot’ (7-point scale; 0 = strongly disagree to 6 = strongly agree) [24]. The last item was reverse coded. To create a sum score, and to ensure that items with different response scales (i.e., 5-point vs. 7-point scale) have equal weighting, we rescaled responses to all items to a scale of 0–1 by multiplying the 5-point response values by ((5/4)/5) and the 7-point response values by ((7/6)/7). The summed score ranged from 0–3 (Cronbach’s alpha = 0.77).
(2) Perceived credibility of FDA as a tobacco regulator was assessed using 15 items from the Credibility of FDA Scale [3] and which appeared to participants in random order. The items tapped into three credibility-related constructs (1) trust (2) expertise, and (3) public interest and used a 5-point Likert scale ranging from 0 = strongly disagree to 4 = strongly agree (See Appendix). Using the same approach described above, the responses were rescaled to a scale of 0–1, and then summed across items (minimum 0, maximum 15) with higher numbers indicating higher credibility of FDA (Cronbach’s alpha = 0.93).
(3) Attitudes toward FDA tobacco regulations was measured by participants’ level of agreement with three statements that appeared in random order: (1) “I think that Americans need to be protected from activities of the tobacco industry”; (2) “It is important that the tobacco industry is regulated”; and, (3) “I support the FDA’s role in protecting Americans’ health from the activities of the tobacco industry” (7-point scale ranging from 0 = strongly disagree to 6 = strongly agree). Responses were rescaled to a scale of 0-1 and then summed across items (minimum 0, maximum 3) with higher numbers indicating more positive attitudes towards FDA regulations (Cronbach’s alpha = 0.84).
(4) Views of the tobacco industry were assessed by ten items adapted from previous studies [26,27,28], and included the following: (1) “Tobacco companies can be trusted to tell the truth”; (2) “Tobacco companies should take responsibility for the harm caused by smoking”; (3) “Tobacco companies have tried to convince the public that there is little or no health risks from secondhand smoke”; (4) “Cigarette companies lie”; (5) “Cigarette companies try to get young people to smoke”; (6) “I would like to see cigarette companies go out of business”; (7) “I would not work for a tobacco company”. The statements appeared in a random order and responses were on 7-point scale; 0 = strongly disagree to 6 = strongly agree. Three additional questions were: (8) “Do tobacco companies give trustworthy information to the public?” (yes, no); (9) “When it comes to the effects of smoking on health, do you think the tobacco companies always, often, sometimes, rarely, or never tell the truth?”; and, (10) “How much do you like tobacco companies?” (5-point scale response, 0 = ‘I do not like them at all’ to 4 = ‘I like them a lot’). Positively worded items were reverse coded. To ensure equal weighting of items with different response scales, responses were first rescaled to a scale of 0–1 and then were summed across items (minimum 0, maximum 10) with higher scores indicating greater negative views of the tobacco industry (Cronbach’s alpha = 0.88).

### 2.4. Sociodemographic and Smoking Related Variables 

At the end of the survey, the participants answered questions about their age, sex, race, education, income, and political orientation. Smokers answered question about their smoking behavior, including questions that assessed their level of nicotine dependence (i.e., number of cigarettes per day and how soon after waking up do they smoke their first cigarette), quit intention, and past quit attempts. 

### 2.5. Statistical Analysis 

All of the analyses were conducted using STATA 13 (StataCorp LCC, College Station, Texas). We began with descriptive statistics to understand the data distribution. Randomization was tested using chi-square and one-way ANOVAs to assess the differences between conditions on various variables. Four-way factorial ANOVAs were carried out to examine the main and interactive effect of each type of information (i.e., FDA’s role, scientific basis, and protective function of regulations) and of smoking status (smoker vs. non-smoker) on each of the four dependent variables: perceived knowledge of FDA roles, credibility of FDA as a tobacco regulator, attitudes toward FDA tobacco regulations, and views of the tobacco industry. We tested for all possible two-way and three-way interactions between the three types of information and for a 4-way interaction between the three types of information and smoking status. Following each ANOVA, the *margins* command in STATA was used to derive marginal predictive group means and standard errors. 

## 3. Results 

### 3.1. Sample Characteristics

Approximately one-half the sample were non-smokers (vs. smokers) and male (Table 2). The majority of participants were of non-Hispanic origin (94%). The race distribution was 83% whites, 6% African Americans, 8% Asian, and 3% of other race groups. Ten percent of participants had a high school level education or less, and 54% had a bachelor’s degree level of education or higher. One-fifth of the sample (20%) reported an annual household income of less than $25,000 and one-fourth of the sample (25%) reported an income of $75,000 or higher.

### 3.2. Randomization Check 

We used chi square (for categorical variables) and one-way ANOVA tests (for continuous variables) to examine the differences between the participants across the eight experimental conditions on various variables that were asked of the full sample. Those included demographic variables (age, gender, race, education, income, and political orientation), and pre-experiment trust in federal government and knowledge of FDA roles. We also examined differences between conditions in smoking-related variables relevant to current smokers (nicotine dependence, desire to quit smoking, quit intention, past quit attempts, number of days smoked in the past month, and average number of cigarettes smoked per day). We observed no statistically significant differences between conditions on any of these variables, suggesting that the random assignment of participants to conditions worked as expected (see results in supplementary Tables S1 and S2). 

### 3.3. Effects of Information Exposure on Outcomes

Four-way factorial ANOVAs were conducted for each of the outcomes. These analyses yielded no statistically significant interactions among the three types of information (i.e., FDA roles, scientific basis of regulations, and protective function of regulations) or between smoking status (smoker vs. non-smoker) and the three types of information. There were several significant main effects for each type of information (see Table 3 for the mean and *SE* for each main effect).

Providing information about FDA’s role increased perceived knowledge of FDA role, F(1, 1765) = 33.9, *p* < 0.001, and improved perceptions of FDA credibility, F(1, 1765) = 13.2, *p* < 0.001, but had no effects on attitudes toward FDA regulations or views of the tobacco industry, Fs(1, 1765) < 2.5, *ps* > 0.10.

Exposure to information about the scientific basis of the FDA’s regulations led to more negative views of the tobacco industry, F(1, 1765) = 6.1, *p* < 0.05, but it had no effects on perceived knowledge, perceptions of FDA credibility, or attitudes toward the regulations, Fs(1, 1765) < 1, *ps* > 0.41.

Providing information about the protective function of FDA regulations served to increase both the credibility of the FDA, F(1, 1765) = 4.9, *p* < 0.05, and the positive attitudes toward FDA regulations, F(1, 1765) = 9.15, *p* < 0.01 but had no effects on perceived knowledge of FDA role or views of the tobacco industry, Fs(1, 1765) < 2.17, *ps* > 0.10.

### 3.4. Effects of Smoking Status on Outcomes

Table 4 presents results on the main effects of smoking status on each of the outcomes. When compared to non-smokers, smokers had higher perceived knowledge of FDA role (M = 1.06 and 1.20, SE = 0.02 and 0.02, respectively), F(1, 1765) = 21.74, *p* < 0.001, lower ratings of FDA credibility (M = 10.42 and 9.61, SE = 0.09 and 0.10, respectively), F(1, 1765) = 36.4, *p* < 0.001, lower support for FDA regulations (M = 2.46 and 2.09, SE = 0.02 and 0.02, respectively), F(1, 1765) = 172.8, *p* < 0.001, and less negative views of the tobacco industry (M = 7.83 and 6.31, SE = 0.06 and 0.06, respectively), F(1, 1765) = 320.2, *p* < 0.001.

## 4. Discussion 

The FDA’s tobacco regulatory and communication framework is responsible for informing the public and various stakeholders about the FDA’s authority over tobacco products and communicating the risks of those products to the public [29]. This study is the first to evaluate the impact of three types of information that characterize the FDA’s tobacco regulatory mission on the perceptions of FDA. These types of information can be broadly used to facilitate understanding of FDA regulations as they demonstrate a positive impact on perceptions of the agency and its tobacco-related regulations.

Information on the FDA’s regulatory roles increased participants’ perceived credibility of FDA. Source credibility, defined as perceived trustworthiness, expertise, and accountability of FDA as a tobacco regulator, is important to the persuasiveness of FDA’s communications about tobacco products, and may influence public and stakeholder’s support for new or existing regulations. Moreover, information on the FDA’s regulatory roles also resulted in a higher perceived knowledge of what the FDA does. Previous research has shown that the knowledge of FDA roles in regulating tobacco is strongly associated with perceived credibility of the organization [11]. Knowledge also plays a role in persuasion and can influence attitude change even in the absence of actual change in information or knowledge [25]. Altogether, these findings suggest that communication strategies that provide information about the tobacco regulatory roles of FDA can increase public awareness and improve the agency’s perceived credibility. 

The FDA bases its regulatory decisions on scientific evidence, and supports scientific research to help explicate impacts of tobacco product use on population health [30]. Interestingly, information on the scientific basis of FDA regulations did not influence perceived credibility of FDA or attitudes toward the regulations. Since the FDA is widely known for regulating and ensuring the safety of food and drug products, it is plausible that participants already knew and perceived FDA as a science-based organization. Though over half of the sample had high levels of education, it is also plausible that participants in the study did not fully understand what it means that regulations are based on scientific evidence. Participants that were exposed to information on the scientific basis of regulations, however, showed a more negative view of the tobacco industry. Perhaps emphasizing the need for rigorous scientific inquiry to support FDA tobacco regulations implies that the FDA is working against an entity that aggressively promotes one of the most lethal products to people’s health—tobacco. 

As a government agency, the extent to which the FDA is perceived to act in the public’s best interest is key to its perceived credibility [4]. Consistent with this idea, we found that information on a protective function of regulation positively influenced the perceptions of FDA credibility and attitudes toward tobacco regulations. This finding is in accord with research that identifies benevolence and agency motives as influential factors on organizational trust [31,32], which is a key determinant of how well risk communications are processed and received by the public [33]. It is important to note that the protective function statement tested in this study referred to protection from presumed promotional and marketing activities of the tobacco industry. Psychological research indicates that harm is mentally represented in terms of perpetrators and victims [34]. From this perspective, a potential protective function of regulations could be designed to protect smokers and nonsmokers from both a harmful product (tobacco) and the agents who promote such a product, thus, involving a consideration of the tobacco industry. Relatedly, Lakoff’s analysis of the concept of regulation indicates that this term highlights the perspective of industry (the body that is regulated) whereas the public perspective involves ‘protections’ rather than ‘regulations’ [35]. It is worth noting that exposure to information on the protective function of regulations, even with reference to industry, did not influence participants’ perceptions of the industry. This protective function statement was also not intended to be used verbatim in any communications; rather, it provides an example of one type of information that FDA could use to inform the public of its tobacco regulatory authority. Multiple potential protective function statements may be equally effective. As FDA’s approach has been to engage the tobacco industry as a stakeholder in dialogues to reduce morbidity and mortality from tobacco use [36], testing out other potential protective function statements for protecting the public from the harmful substances in tobacco products is essential. 

Overall, the differences between participants that were exposed to information on FDA regulatory mission and controls were significant, but small. The differences are impressive, however, given the one-time brief online exposure to an informative statement about FDA. It is important to note that the effect of each informational statement on the perceptions of the FDA was not moderated by smoking status. Thus, the informational statements influenced both smokers and non-smokers equally. Some differences between smokers and nonsmokers were observed, however. Smokers perceived FDA as less credible than did nonsmokers and they reported weaker attitudes in favor of tobacco regulations. Smokers also reported less negative views of the tobacco industry. Such differences are not surprising. Previous research has demonstrated lower levels of trust among smokers in sources of health information [37] and in government agencies [5]. In addition, research on information processing shows that when a message is threatening to one’s existing attitude, those who believe that the message is relevant to them are more likely to generate critical judgments regarding the message’s content, quality, and credibility than individuals for whom the message is less relevant [38,39]. In this context, it is particularly heartening that informational messages detailing the role, scientific basis, and a potential protective function of tobacco regulations changed smokers’ perceptions of the FDA.

## 5. Limitations 

The current study has several limitations. The study used a convenience sample of MTurk workers; hence, the findings cannot be generalized to the general US population. Though demographic characteristics of MTurk samples (i.e., higher representation of highly educated participants) do not resemble the demographic characteristics of nationally representative samples, previous studies have shown that MTurk is a valid recruitment tool for experimental studies on tobacco-related issues allowing researchers to minimize threats to internal validity [40]. Moreover, studies that compared results from MTurk samples to results from nationally representative samples show comparable experimental and correlational results [41,42]. Since this is the first study to test types of information to improve the perceptions of FDA, the findings are preliminary and should be further investigated in nationally representative samples of Americans to better inform FDA educational efforts. Although data were self-reported, hence they are subject to related biases, such as recall and social desirability biases, we used attention checks to gauge participants’ attention to and compliance with study instructions; participants who failed these checks (*n* = 197) were excluded from the analysis. Lastly, we did not assess participants’ risk perceptions concerning use of tobacco products post exposure to information on FDA as a tobacco regulator. Future research will need to determine whether providing information about the FDA’s role, function, and the basis of its regulations influences the risk perceptions of tobacco use. 

## 6. Conclusions 

Public perceptions of the FDA’s tobacco regulations may influence the public and stakeholder receptivity to FDA tobacco communications and compliance with tobacco regulations. This study provides experimental evidence that casting the FDA tobacco regulatory mission in a frame that includes explaining the FDA regulatory roles, scientific basis of regulations, and the possible protective functions of regulations, may improve public perceptions of FDA and increase source credibility. FDA and other tobacco prevention organizations may examine this framework to determine how they may better communicate to the public various missions that are aimed at reducing tobacco-related diseases and protecting the public health. 

## Figures and Tables

**Table 1:** Information types and their corresponding statements in a 2 × 2 × 2 between subjects experimental design.

				Protective Function of Regulations	
				Yes	No
**FDA Roles**	Yes	**Scientific Basis of Regulations**	Yes	1. The Food and Drug Administration (FDA) regulates the manufacture, marketing, and distribution of tobacco to safeguard people’s health. FDA regulations are based on scientific evidence, and are designed to protect Americans from the activities of the tobacco industry.	2. The Food and Drug Administration (FDA) regulates the manufacture, marketing, and distribution of tobacco to safeguard people’s health. FDA regulations are based on scientific evidence.
			No	3. The Food and Drug Administration (FDA) regulates the manufacture, marketing, and distribution of tobacco to safeguard people’s health. FDA regulations are designed to protect Americans from the activities of the tobacco industry.	4. The Food and Drug Administration (FDA) regulates the manufacture, marketing, and distribution of tobacco to safeguard people’s health.
	No	**Scientific Basis of Regulations**	Yes	5. Regulations of the Food and Drug Administration (FDA) are based on scientific evidence, and are designed to protect Americans from the activities of the tobacco industry.	6. Regulations of the Food and Drug Administration (FDA) are based on scientific evidence.
			No	7. Regulations of the Food and Drug Administration (FDA) are designed to protect Americans from the activities of the tobacco industry.	8. None

**Table 2:** Sample demographic characteristics (N = 1766).

Variables	% or Mean (SD)
**Smoking status**	
Non-smoker	51
Smoker	49
**Age (18–75)**	35 (11.6)
**Sex**	
Female	50
Male	50
**Hispanic ethnicity**	
No	94
Yes	6
**Race**	
White	83
African American	6
Asian	8
Other	3
**Education**	
High school or less	10
Some College	24
Associate degree	12
Bachelor’s degree or higher	54
**Annual household income**	
Below $25,000	19
$25,000–$49,999	30
$50,000–$74,999	25
$75,000 or higher	26

**Table 3:** Impact of Information Exposure on Outcomes.

Dependent Variables	Information about FDA role in Regulating Tobacco		Information about Scientific Basis of FDA Regulations		Information about Protective Function of FDA Regulations	
	Presented	Control	Presented	Control	Presented	Control
Perceived knowledge of FDA ^a^	1.21 (0.02) ***	1.04 (0.02)	1.14 (0.02)	1.12 (0.02)	1.15 (0.02)	1.11 (0.02)
Credibility of FDA ^a^	10.27 (0.09) ***	9.77 (0.10)	10.02 (0.09)	10.03 (0.10)	10.17 (0.09) *	9.87 (0.10)
Attitudes toward FDA regulations ^a^	2.31 (0.02)	2.26 (0.02)	2.28 (0.02)	2.28 (0.02)	2.33 (0.02) **	2.24 (0.02)
View of the tobacco industry ^b^	7.08 (0.06)	7.10 (0.06)	7.20 (0.06) *	6.98 (0.06)	7.16 (0.06)	7.02 (0.06)

Note. Values are means and standard errors (SE); * *p* < 0.05, ** *p* < 0.01, *** *p* < 0.001; ^a^ higher numbers indicate higher perceived knowledge, higher credibility, and stronger positive attitudes toward tobacco regulations; ^b^ higher numbers indicate stronger negative views of the tobacco industry.

**Table 4:** Impact of Smoking Status on Outcomes.

Dependent Variables	Non-Smokers	Smokers
	Mean (SE)	Mean (SE)
Perceived knowledge of FDA ^a^	1.06 (0.02)	1.20 (0.02) ***
Credibility of FDA ^a^	10.42 (0.09)	9.61 (0.10) ***
Attitudes toward FDA regulations ^a^	2.46 (0.02)	2.09 (0.02) ***
View of the tobacco industry ^b^	7.83 (0.06)	6.31 (0.06) ***

Note. *** *p* < 0.001; ^a^ higher numbers indicate higher perceived knowledge, higher credibility, and more positive attitudes toward tobacco regulations; ^b^ higher numbers indicate more negative views of the tobacco industry.

## Supplementary Materials

The following are available online at http://www.mdpi.com/1660-4601/15/4/753/s1, Table S1: Chi square test and one-way analysis of variance (ANOVA) examining differences between experimental conditions on demographics, trust in government, and knowledge of FDA roles, Full sample (n = 1766), and Table S2: Chi square test and one-way analysis of variance (ANOVA) examining differences between experimental conditions on smoking related variables, subsample of current smokers (n = 863).

## Appendix

**FDA tobacco credibility scale—FDA TCS^3^**
1. The FDA knows about the risks of tobacco use
2. Information from the FDA about tobacco use is written by experts
3. The FDA has the expertise to communicate about the risks of tobacco products to the public
4. The FDA is an expert at making rules about the harms of tobacco use
5. The FDA has experience making sure rules about tobacco products are followed
6. The FDA is interested in the public’s well-being when it makes rules about tobacco product advertising
7. The FDA cares about the public when it limits how tobacco companies can market tobacco products
8. The FDA has my interest in mind when it makes rules about the manufacturing of tobacco products
9. The FDA is watching out for the public by limiting how cigarettes can be sold
10. The FDA treats the public with respect when it makes rules about tobacco products
11. Information from the FDA about tobacco products is fair and balanced
12. I trust the FDA to protect people from tobacco advertising
13. The FDA is capable of limiting tobacco product marketing
14. I trust the FDA to make sure its rules about advertising tobacco products are followed
15. The FDA knows how to keep tobacco companies from showing misleading advertisements
Note. Response options were on 5-point Likert agreement scale ranging from 1 = Strongly disagree to 5 = Strongly agree.
