# Supplementary material for: Information to Improve Public Perceptions of the Food and Drug Administration (FDA’s) Tobacco Regulatory Role
Source: Int J Environ Res Public Health. 2018 Apr 14;15(4):753. doi: 10.3390/ijerph15040753 (PMC5923795; doi:10.3390/ijerph15040753)
Supplement: Supplementary file 1 [file ijerph-15-00753-s001.pdf]

Table S1. Chi square test and one-way analysis of variance (ANOVA) examining differences between experimental conditions on demographics, trust in government, and knowledge of FDA roles, Full sample (n = 1766).

	Condition										
	1	2	3	4	5	6	7	8	Total	Pearson chi2 or F statistic	P value
	%	%	%	%	%	%	%	%	%		
**Smoking status**											
Non-smoker	50.65	51.77	49.53	46.33	55.16	50.9	50	55.14	51.19	5.22	0.632
Smoker	49.35	48.23	50.47	53.67	44.84	49.1	50	44.86	48.81		
Total	100	100	100	100	100	100	100	100	100		
**Age**											
Mean (SD), Range 18-75	36.17 (12.4)	34.4 (11.1)	34.52 (11.2)	36.28 (12.2)	36.18 (12.5)	34.99 (11.9)	34.78 (11.6)	35.46 (11.7)	35.35 (11.9)	0.97	0.455
**Sex**											
Male	53.25	50	49.53	52.75	47.09	50	46.85	48.83	49.8	3.38	0.847
Female	46.75	50	50.47	47.25	52.91	50	53.15	51.17	50.2		
Total	100	100	100	100	100	100	100	100	100		
**Hispanic origin**											
No	93.94	93.36	93.46	94.95	91.93	94.59	91.44	95.33	93.62	4.95	0.666
Yes	6.06	6.64	6.54	5.05	8.07	5.41	8.56	4.67	6.38		
Total	100	100	100	100	100	100	100	100	100		
**Race**											
White	80.09	80.97	81.78	85.32	80.72	84.23	85.14	83.64	82.71	20.98	0.462
African American	8.23	5.31	6.54	4.13	9.42	5.41	4.05	7.48	6.33		
Asian	7.36	9.73	7.94	8.26	8.97	9.01	7.66	6.54	8.19		
Other race	4.33	3.98	3.74	2.29	0.9	1.35	3.15	2.34	2.77		
Total	100	100	100	100	100	100	100	100	100		
**Education**											
High school or less	12.12	8.85	7.48	11.47	10.76	9.46	9.91	10.28	10.06	15.56	0.793
Some college	22.94	27.88	22.43	25.69	21.52	26.13	27.03	20.09	24.24		
Associate degree	11.26	12.83	10.28	9.63	13.9	13.96	12.16	14.95	12.37		
Bachelor's degree or higher	53.68	50.44	59.81	53.21	53.81	50.45	50.9	54.67	53.33		
Total	100	100	100	100	100	100	100	100	100		
**Household annual income**											
Below $25,000 per year	16.45	21.68	18.69	19.82	13.9	19.37	18.02	21.5	18.65	22.78	0.744
Between $25,000 and $49,999 per year	32.03	30.97	29.44	25.35	34.08	25.68	29.28	30.84	29.73		

Between $50,000 and $74,999	25.11	25.22	25.23	29.03	23.77	26.13	25.68	21.96	25.27		
$75,000 or more	25.97	22.12	25.23	23.96	26.91	26.58	26.13	25.23	25.27		
Unknown income	0.43	0	1.4	1.84	1.35	2.25	0.9	0.47	1.07		
Total	100	100	100	100	100	100	100	100	100		
**Political orientation: Here is an 11-point-scale on which people's political views are arranged from extremely liberal to extremely conservative. Where would you place yourself on this scale?”**											
Mean (SD), Range 1-11	4.70 (2.7)	5.38 (2.8)	4.68 (2.8)	4.83 (2.8)	4.88 (2.7)	5.00 (3.0)	5.18 (3.0)	5.27 (2.9)	4.99 (2.9)	1.85	0.0745
**Trust in federal government: How much trust do you have in the Federal government?** [a]											
None at all	11.26	12	9.81	12.96	11.21	17.19	11.31	11.32	12.14	24.75	0.258
A little	44.16	43.56	45.33	35.19	48.43	36.2	41.63	45.75	42.54		
A fair amount	37.23	32.44	35.05	39.81	31.84	38.46	39.37	35.85	36.25		
A great deal	7.36	12	9.81	12.04	8.52	8.14	7.69	7.08	9.08		
Total	100	100	100	100	100	100	100	100	100		
**FDA knowledge: Do you think the FDA regulates how cigarettes and other tobacco products are made?** [a]											
No	26.84	26.99	30.37	23.85	29.6	35.59	24.77	29.44	28.42	16.98	0.257
Yes	61.04	56.19	55.14	62.84	56.5	48.65	57.66	53.27	56.44		
I do not know	12.12	16.81	14.49	13.3	13.9	15.77	17.57	17.29	15.14		
Total	100	100	100	100	100	100	100	100	100		
**FDA knowledge: Do you think the FDA regulates how cigarettes and other tobacco products are advertised?** [a]											
No	12.12	21.24	14.02	13.76	18.83	14.86	16.22	15.42	15.82	17.74	0.219
Yes	84.42	73.89	80.84	81.19	73.54	77.48	76.58	78.04	78.25		
I do not know	3.46	4.87	5.14	5.05	7.62	7.66	7.21	6.54	5.93		
Total	100	100	100	100	100	100	100	100	100		
**FDA knowledge: Do you think the FDA regulates how cigarettes and other tobacco products are sold in stores?** [a]											
No	11.89	23.87	20.09	17.43	18.18	19.46	15.45	15.64	17.74	18.54	0.183
Yes	80.62	68.47	72.9	76.61	71.82	70.59	75.45	74.88	73.93		
I do not know	7.49	7.66	7.01	5.96	10	9.95	9.09	9.48	8.33		
Total	100	100	100	100	100	100	100	100	100		
**FDA knowledge: Do you think the FDA communicates the risks of cigarettes and other tobacco products to the public?** [a]											
No	9.52	20.8	19.16	14.22	17.49	20.27	16.67	20.09	17.23	22.09	0.077
Yes	84.85	73.01	76.64	83.03	78.48	73.87	77.03	74.3	77.68		
I do not know	5.63	6.19	4.21	2.75	4.04	5.86	6.31	5.61	5.08		
Total	100	100	100	100	100	100	100	100	100		
**FDA knowledge: Do you think the FDA regulates the amount of tobacco that can be grown and imported?** [a]											
No	32.47	38.05	33.64	35.32	35.87	36.49	32.13	32.86	34.62	8.54	0.859
Yes	37.23	32.3	33.18	34.86	37.67	34.23	31.67	34.74	34.5		

I do not know	30.3	29.65	33.18	29.82	26.46	29.28	36.2	32.39	30.88
Total	100	100	100	100	100	100	100	100	100
	32.47	38.05	33.64	35.32	35.87	36.49	32.13	32.86	34.62

Note: Numbers are percentages and Pearson chi square for categorical variables, and means, standard deviation, f statistics for continuous variables. [a] questions were asked at pretest.

Table S2. Chi square test and one-way analysis of variance (ANOVA) examining differences between experimental conditions on smoking related variables, subsample of current smokers (n=863).

	**Condition**										
	**1**	**2**	**3**	**4**	**5**	**6**	**7**	**8**	**Total**	**Pearson chi2 or F statistic**	**P value**
	%	%	%	%	%	%	%	%	%		
**Nicotine dependence: How soon after waking do you usually have your first cigarette?**											
Within the first 5 minutes	14.04	15.6	14.02	24.14	22	9.17	10.81	8.42	14.87	27.74	0.147
6-30 minutes	30.7	24.77	30.84	24.14	27	33.03	39.64	35.79	30.66		
31-60 minutes	17.54	20.18	21.5	19.83	19	19.27	18.02	15.79	18.93		
More than 60 minutes	37.72	39.45	33.64	31.9	32	38.53	31.53	40	35.54		
Total	100	100	100	100	100	100	100	100	100		
**Desire to quit smoking: How much do you want to quit smoking?**											
Not at all or a little	32.46	33.94	38.89	33.62	31	29.36	23.42	43.16	33.06	17.19	0.246
Moderately	29.82	29.36	22.22	28.45	32	33.03	26.13	27.37	28.54		
Very or extremely	37.72	36.7	38.89	37.93	37	37.61	50.45	29.47	38.4		
Total	100	100	100	100	100	100	100	100	100		
**Quit intention: Are you planning to quit smoking?**											
Within the next month or 6 months	42.11	37.61	46.3	43.97	42	37.61	54.05	33.68	42.34	11.97	0.101
Beyond 6 months or not at all	57.89	62.39	53.7	56.03	58	62.39	45.95	66.32	57.66		
Total	100	100	100	100	100	100	100	100	100		
**Quit attempts: Have you made any attempts to stop smoking in the past?**											
No	50.88	56.88	61.11	56.9	54	51.38	47.75	57.89	54.52	5.94	0.546
Yes	49.12	43.12	38.89	43.1	46	48.62	52.25	42.11	45.48		
Total	100	100	100	100	100	100	100	100	100		
**Number of days smoked: In the past 30 days, on how many days did you smoke cigarettes?**											
0-10	23.68	27.52	22.22	21.55	22	16.51	18.92	27.37	22.39	20.77	0.107
11-20	13.16	13.76	23.15	12.93	8	19.27	21.62	11.58	15.55		
21+	63.16	58.72	54.63	65.52	70	64.22	59.46	61.05	62.06		
Total	100	100	100	100	100	100	100	100	100		
**Number of Cigarettes per day: On average, how many cigarettes do you smoke each day?**											

Mean (SD), Range 0-100	7.88 (7.8)	7.74 (7.9)	8.48 (8.6)	10.9 (12.0)	8.89 (9.0)	7.12 (7.6)	7.74 (6.8)	8.34 (8.3)	8.29 (8.2)	1.40	0.2002

Note: Numbers are percentages and Pearson chi square for categorical variables, and means, standard deviation, f statistics for continuous variables.

© 2018 by the authors. Submitted for possible open access publication under the terms and conditions of the Creative Commons Attribution (CC BY) license (http://creativecommons.org/licenses/by/4.0/).
